# Differential Modulation of Glutamate Transporter‐1 by Cocaine and Oxycodone and the Efficacy of MC‐100093 to Reduce Reinstatement of Self‐Administration

**DOI:** 10.1002/brb3.70616

**Published:** 2025-07-10

**Authors:** Damyan W. Hart, Yanaira Alonso‐Caraballo, Britta Hornback, Angel Robert, Megan A. Brickner, Manuel Esguerra, Wayne E. Childers, Magid Abou‐Gharbia, Mark J. Thomas

**Affiliations:** ^1^ Department of Neuroscience University of Minnesota Minneapolis Minnesota USA; ^2^ Medical Discovery Team on Addiction University of Minnesota Minneapolis Minnesota USA; ^3^ Graduate Program in Neuroscience University of Minnesota Minneapolis Minnesota USA; ^4^ School of Pharmacy Temple University Philadelphia Pennsylvania USA

## Abstract

**Introduction:**

Despite the widespread impact of opioid use disorder, pharmacological options for treatment remain limited. Recent studies find that cocaine exposure decreases the expression of the glutamate transporter GLT‐1 in the nucleus accumbens (NAc) and that treatment with the beta‐lactam antibiotic ceftriaxone rescues this loss of expression and reduces cue‐induced reinstatement to cocaine self‐administration. The novel beta‐lactam derivative MC‐100093 (093) lacks antimicrobial properties but crosses the blood‐brain barrier more rapidly and retains the beneficial effects of ceftriaxone following cocaine. However, 093 effects following oxycodone exposure have not been examined.

**Methods:**

We used intravenous self‐administration (IVSA) of oxycodone in rats to test if 093 can attenuate oxycodone seeking. Membrane expression of GLT‐1 in the NAc was investigated using western blots. Conditioned place preference (CPP) was used to test the effect of oxycodone and cocaine alone on GLT‐1 expression.

**Results:**

We find that 093 injections following IVSA of oxycodone in rats did not reduce cue‐induced reinstatement. Interestingly, western blot analysis revealed that 093 failed to upregulate the expression of GLT‐1 in the NAc of oxycodone‐exposed animals. Follow‐up CPP experiments suggest that oxycodone exposure alone does not decrease GLT‐1 expression as cocaine does.

**Conclusions:**

Our results indicate that drug‐specific reductions in NAc GLT‐1 expression may be necessary for 093's efficacy. Further investigation into 093 and other opioids is needed to fully understand their relationship with GLT‐1 expression and beta‐lactams.

## Graphical abstract

The beta lactam derivative MC‐100093 (093) did not attenuate reinstatement of oxycodone seeking following extinction of self‐administration. In addition, 093 did not upregulate membrane expression of glutamate transporter 1 (GLT‐1) in the nucleus accumbens. Follow‐up conditioned place preference experiments revealed that drug specific modulation of GLT‐1 expression may underlie the efficacy of 093 to reduce reinstatement. Created in BioRender. Hart, D. (2025) https://BioRender.com/w58u294.

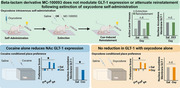

## 1 Introduction

The widespread prescription of OxyContin (oxycodone) as a non‐addictive opioid has heavily contributed to the rise of opioid use disorder (OUD) (Alpert et al. 2022). Unfortunately, pharmacological treatment options are limited for oxycodone and other OUDs and those that are approved by the U.S. Food and Drug Administration (FDA) are opioids themselves. Namely, treatments such as methadone and buprenorphine rely on extended activation of mu‐opioid receptors to reduce craving in an effort to prevent relapse (Volkow and Blanco 2023). These treatments have significant limitations to their access and ease of prescription. There exists an urgent need for novel pharmacological tools to reduce the likelihood of relapse and help in the recovery path for those suffering from OUD.

A number of theories of addiction offer potential therapeutic avenues for pharmacological targets. One such theory, the glutamate hypothesis, emerged in the early 2000s and postulates that increased glutamate signaling from the prefrontal cortex to the nucleus accumbens (NAc) drives relapse‐like behavior (McFarland et al. 2003). Around the same time, a screen of FDA‐approved drugs identified the beta‐lactam antibiotic ceftriaxone as a positive modulator of GLT‐1 expression (Rothstein et al. 2005). GLT‐1 is an astrocytic glutamate transporter responsible for the rapid clearance of glutamate from the synaptic cleft (Tanaka et al. 1997). Since then, it has been repeatedly shown in preclinical models of SUD that GLT‐1 expression is reduced in the NAc following exposure to cocaine, thereby increasing glutamate availability (Knackstedt et al. 2010; Smaga et al. 2020). Subsequent treatment with ceftriaxone rescues this decrease in expression and reduces cue‐induced reinstatement following extinction of cocaine self‐administration. However, a barrier to the translational potential of this work are increased risks of antibiotic‐resistant bacterial strains appearing with widespread antibiotic treatment (Tacconelli et al. 2009). To curb this, a novel beta‐lactam derivative, MC‐100093 (093), was developed. This compound lacks the anti‐microbial properties of other beta‐lactams such as ceftriaxone but recapitulates its ability to rescue GLT‐1 expression and reduce cue‐induced reinstatement for cocaine‐seeking (Knackstedt et al. 2021).

Most of the preclinical work with beta‐lactams and SUDs has focused on cocaine and other psychostimulants (Smaga et al. 2020). However, with the escalation of OUD prevalence, it is of interest to investigate this phenomenon with opioids as well. Following heroin self‐administration, NAc GLT‐1 expression is reduced along with deficits in glutamate uptake (Shen et al. 2014). Treatment with ceftriaxone rescued the heroin‐induced changes in glutamate dynamics and attenuated cue‐induced reinstatement in a GLT‐1‐dependent manner. Repeated exposure to morphine reduces NAc GLT‐1 expression which is restored with ceftriaxone treatment (Sari et al. 2024). Our lab has also shown that following morphine exposure ceftriaxone rescues abstinence‐dependent synaptic changes in the NAc and prevents reinstatement of morphine conditioned place preference (CPP) (Hearing et al. 2016). Overall, reports generally support the efficacy of ceftriaxone in attenuating opioid‐induced behavioral and synaptic phenotypes. However, to our knowledge, no studies to date have centered on beta‐lactams and oxycodone. The goal of this study was to investigate whether the novel beta‐lactam derivative MC‐100093 reduces cue‐induced reinstatement to oxycodone following the extinction of self‐administration.

## 2 Materials and Methods

### 2.1 Animals

Nineteen (10 male and 9 female) adult Long Evans rats were purchased from Charles River Laboratory (Wilmington, MA). Rats were housed in specific pathogen‐free conditions on a 12 h/12 h light/day cycle using Teklad (West Lafayette, IN) corn cob bedding and Teklad 2918 feed ad libitum. Male rats were 210–370 g and female rats 150–240 g at the initiation of catheter surgeries. Thirty‐six (18 male and 18 female) C57BL/6J adult mice were purchased from Jackson Laboratory (Bar Harbor, ME). Mice were 6–8 weeks old at the beginning of CPP experiments. Mice were housed in a conventional setting on a 12 h/12 h light/day cycle in Teklad corn cob bedding and Teklad 2918 feed ad libitum. All experiments involving animals were approved by the University of Minnesota Institutional Animal Care and Use Committee.

### 2.2 Intravenous Self‐Administration

IVSA studies were run similar to previous studies (Mavrikaki et al. 2017). Surgeries for jugular catheter implant (SAI infusions, RSB‐SA‐7.5CF and RSB‐SA‐7.5CM; Lake Villa, IL) were done under isoflurane anesthesia (1%–5%). Catheters were flushed daily with heparin/saline mixture (0.2 mL; 30 units/ml; I.V.) and twice weekly with gentamicin (0.2 mL; 10 mg/ml; I.V.). Catheter patency was confirmed with intravenous Brevital (0.1 mL females; 0.2 males of 10 mg/ml I.V.) before the self‐administration experiment began and at the beginning of extinction. Intravenous oxycodone self‐administration (IVSA) was carried out in single chamber Med Associates boxes (Fairfax, VT) on a fixed ratio 1 (FR1) schedule of reinforcement. Two levers with respective cue lights and a house light on the opposing wall were present. One lever was assigned as the active lever and the other as the inactive lever. At the beginning of the session, the house light turned on. When the active lever was pressed the house light turned off, the associated cue light turned on for 10 s, and 0.01 mg/kg oxycodone per infusion was delivered over 4 s through the intravenous catheter. This dose was selected based on previous dose‐response curves finding 0.01 mg/kg/infusion resulted in the highest number of infusions between a range of 0.0003 and 0.3 mg/kg/infusion (Mavrikaki et al. 2017; Hinds et al. 2023). During the 10 s, active lever presses were recorded but did not initiate additional drug delivery. Once the cue light turned back on, the active lever could again be pressed for drug delivery. Pressing of the inactive lever did not turn off the house light, did not turn on its associated cue light, and no drug was delivered. Rats began with 3 days of short access (1 h/day) sessions to learn the task before moving to 10 days of long access (4 h/day). After long access, rats began 10 days of extinction (2 h/day). During extinction, pressing the active lever no longer turned on its associated cue light, nor turned off the house light, and no drug was delivered. After each of the final six extinction sessions, rats were injected intraperitoneally (i.p.) with 50 mg/kg MC‐100093 (093) or saline. This dose of 093 has previously been demonstrated to be the most efficacious at modulating NAc GLT‐1 expression in rats (Knackstedt et al. 2021). Rats were assigned to either the saline or 093 group by ranking them based on the total number of active lever presses during long access and then equally splitting them into two balanced groups. After the final extinction session, rats underwent a single 2‐h cue‐induced reinstatement session. During reinstatement, pressing the active lever turned on its associated cue light and turned off the house light but the drug was not delivered. Reinstatement active lever pressing was compared to the average lever pressing of the last 3‐days of extinction. A MATLAB (MathWorks; Natick, MA) script was written to analyze within‐session active lever pressing from Med Associates timestamps.

### 2.3 CPP for Cocaine and Oxycodone

Mouse CPP experiments were performed using a 25 × 45 cm two‐chamber apparatus constructed in‐house. One of the chambers had mesh flooring with the center paneling painted white while the other had rod flooring with the center paneling painted black. No other spatial cues were present. The CPP protocol entailed two days of preference pre‐test, followed by eight days of conditioning, and a preference test on the following day. Animals then underwent seven days of home cage‐forced abstinence with daily saline injections followed by a final preference retest. This protocol was previously used to identify differences in GLT‐1 following CPP for cocaine (Niedzielska‐Andres et al. 2019). During the pretest, animals were injected with 0.1 mL/kg of saline and placed in their CPP box for 20 min. A biased design was selected as we expected the development of a preference for the drug‐paired side. For the biased design, the conditioned stimulus side (CS+) was assigned to the side in which the animal spent the least amount of time on average during the two days of the pretest. Conditioning was done by alternating days of injecting the conditioned stimulus (15 mg/kg cocaine; 3 mg/kg oxycodone) in the assigned CS+ side or saline on the CS‐ side for 30 min. After four pairings across eight days, animals’ preference was tested. During this session, the barrier separating entry between the two sides was removed, and after saline injection, animals were free to roam the apparatus for 20 min. The preference score was calculated by finding the difference between times spent on the conditioned side versus the unconditioned side. The retest session was identical to the preference test.

### 2.4 CPP for MC‐100093

The general methods for the 093 CPP (50 mg/kg 093 on CS+ days) were the same as those for cocaine and oxycodone except for two differences. First, an unbiased design was used as it was not known whether the compound would produce a preference or aversion to the paired side. Second, these animals did not undergo a seven‐day forced home cage abstinence and subsequent retest. That was done in order to replicate a study to identify changes in GLT‐1 expression, while this experiment's goal was to see specifically whether 093 generated a preference or aversion to the paired side. The chosen dose of 093 (50 mg/kg) has previously been used in studies with mice (Hinds et al. 2023).

### 2.5 Tissue Homogenization and Fractionation

Twenty‐four hours after IVSA reinstatement or the final CPP test, animals were deeply anesthetized with isoflurane and rapidly decapitated. Both the left and right NAc were dissected out and immediately frozen at −80°C until further processing. For processing, frozen tissue was placed immediately into freshly made ice‐cold homogenization buffer: 320 mM sucrose, 4 mM HEPES, 1:100 HALT Protease + Phosphatase Inhibitors (Thermo Scientific 78440; Waltham, MA), 1:100 EDTA, and 10 mM DTT. 500 uL of homogenization buffer was used for the rat accumbens tissue while 200 uL of buffer was used for the mouse tissue. Tissue was homogenized with the following repetitive passages with a 1 mL insulin syringe: 3x no needle, 5x‐18G, 5x‐22G, and 5x‐25G. Lysates subsequently underwent sequential microcentrifugation to produce a membrane‐enriched fraction, as previously described (Knackstedt et al. 2010, 2021). In brief, homogenized lysates were initially spun at 1600 × *g* for 5 min. Supernatants were removed and placed in a new tube while pellets (P1) were saved and frozen. The supernatants were spun again at 10k RPM for 10 min. The resulting supernatant (S1) was saved and frozen. The pellet was resuspended with 200 uL of tissue buffer (320 mM sucrose and 4 mM HEPES) and spun a final time at 11k RPM for 15 min. The final supernatant (S2) was removed and frozen. Bulk rat forebrain tissue was used for protocol validation and the final pellet (P2) was reconstituted with RIPA buffer (Thermo Scientific 89900) with an additional 0.9% SDS (final concentration of 1.0% SDS). All spins were performed at 4°C in a Microfuge 20R with an FA241.5P rotor (Beckman Coulter; Brea, CA). To test the hypothesis that RIPA buffer was in part responsible for the presence of higher molecular weight GLT‐1 bands, we homogenized and fractionated a separate set of lysates. Before the final spin to generate the P2 pellet, each lysate was split in half to examine how different reconstitution buffers affected multimerization from the same source sample. The two sets went through the final spin and following removal of the supernatant, one set of the P2 pellets was reconstituted with RIPA buffer + 1% SDS while the other was reconstituted with a standard tissue buffer (320 mM sucrose + 4 mM HEPES) + 1% SDS. The two sets of P2 lysates were examined by western blot and probed for GLT‐1 and calnexin, another membrane protein more often used in GLT‐1 studies (Fujio et al. 2005). We also tested if boiling the samples before loading the gel was exacerbating this phenomenon, as this behavior has been previously described for GLT‐1 (Zou et al. 2011).

### 2.6 Western Blot for GLT‐1

The final membrane fraction for experimental samples was not reconstituted with RIPA buffer but rather with a standard tissue buffer (320 mM sucrose and 4 mM HEPES) + 1% SDS. The protein concentration was determined with the Pierce BCA Protein Assay Kit (Thermo Scientific 23227). To assess changes in GLT‐1 with SDS‐PAGE, 5 uL of Laemmli loading buffer (Invitrogen LC2676; Waltham, MA) with β‐ME (1:100) were added to 7 µg of protein lysate and loaded into 15‐well Novex 10% Tris‐Glycine gels (Invitrogen XP00105BOX). Importantly, experimental samples were not heated to 95°C prior to loading the gel which was run for 2.5 h at 80 V. Protein was then transferred for 2 h onto nitrocellulose membranes (Thermo Scientific 88025) at 0.4A. After blocking for 1 h with 5% BSA (Tocris 52–171; Bristol, UK) in TBS + 0.1% Tween‐20 (TBST), membranes were incubated with GLT‐1 (Sigma‐Aldrich AB1783; Burlington, MA) and calnexin (Enzo ADI‐SPA‐860; Farmingdale, NY) or pan‐cadherin (Cell Signaling 4073; Danvers, MA) primary antibodies overnight at 4°C. The following day, membranes were washed with 5 × 5 min of TBST. Membranes were subsequently incubated again with secondary antibodies donkey α guinea pig IR800 (LI‐COR 926–32411; Lincoln, NE) and goat α rabbit IR680 (LI‐COR 926–68071) for 1 h in 5% BSA in TBST at room temperature. Following 5 × 5 min washes with TBST, membranes were imaged on a LI‐COR Odyssey and quantified with ImageJ (NIH; Bethesda, MD). GLT‐1 signal was normalized to each sample's respective calnexin signal. A subset of animals (*n* = 7) in the oxycodone IVSA experiment did not have their tissue collected within 24 h of the cue‐induced reinstatement session and were not included in the western blot analyses. All mice in the cocaine and oxycodone CPP cohorts had their tissue collected within the 24‐h time frame of the final preference retest and were included in the western blot studies.

### 2.7 Statistical Analysis

All statistical tests were done in JMP (JMP Statistical Discovery; Cary, NC). Normal distributions were tested with the Shapiro–Wilk test and equal variances with the Levene test. For experiments with non‐normal distributions, unequal variances, or small/uneven sample sizes, the Wilcoxon exact test was used. One‐factor comparisons that satisfied parametric assumptions were analyzed with Student's *t*‐test. One‐factor analyses with multiple comparisons were done with Bonferroni corrections. Experiments involving more than two repeated measurements were analyzed by mixed models. Repeated measurement experiments with only two observation points that satisfied parametric assumptions were analyzed with paired Student's *t*‐test. Due to limited group sizes when split by sex, sex as a variable was only tested for in the IVSA study. Complete statistical tests can be found in Table 1.

**TABLE 1:** All results from statistical tests performed in the analysis of the data in this study.

Figure	Comparison	Test	Results	Sample size
1B	Short access active vs. inactive lever	Mixed model	Day F(2,72) = 2.371, *p* = 0.1006; Lever F(1,36) = 4.619, *p* = 0.0384; Day*lever F(2,72) = 1.703, *p* = 0.1894	*n* = 4 oxycodone/saline males *n* = 5 oxycodone/saline females *n* = 6 oxycodone/093 males *n* = 4 oxycodone/093 females
	Long access active vs. inactive lever	Mixed model	Day F(9,324) = 1.0953, *p* = 0.3657; Lever F(1,36) = 13.0907, *p* = 0.0009; Day*lever F(9,324) = 0.7578, *p* = 0.6556	
	Extinction active vs. inactive lever	Mixed model	Day F(3,108) = 1.5051, *p* = 0.2174; Lever F(1,36) = 1.1990, *p* = 0.2808; Day*lever F(3,108) = 0.0345, *p* = 0.9914	
	Extinction + treatment active vs. inactive lever	Mixed model	Day F(5,180) = 1.4825, *p* = 0.1976; Lever F(1,36) = 0.0700, *p* = 0.7929; Day*lever F(5,180) = 0.4943, *p* = 0.7802	
1C	Short access Oxy/Sal vs. Oxy/093 active lever presses	Mixed model	Day F(2,34) = 2.3473, *p* = 0.1109; Treatment F(1,17) = 0.0081, *p* = 0.9295; Day*treatment F(2,34) = 0.2163, *p* = 0.8066	
	Long access Oxy/Sal vs. Oxy/093 active lever presses	Mixed model	Day F(9,153) = 0.9429, *p* = 0.4899; Treatment F(1,17) = 1.5782, *p* = 0.2260; Day*Treatment F(9,153) = 0.5810, *p* = 0.8112	
	Extinction Oxy/Sal vs. Oxy/093 active lever presses	Mixed model	Day F(3,51) = 0.7761, *p* = 0.5127; Treatment F(1,17) = 0.6422, *p* = 0.4340; Day*treatment F(3,51) = 0.0899, *p* = 0.9652	
	Extinction + treatment Oxy/Sal vs. Oxy/093 active lever presses	Mixed model	Day F(5,85) = 2.645, *p* = 0.0285; Treatment F(1,17) = 0.6563, *p* = 0.4291; Day*treatment F(5,85) = 1.3874, *p* = 0.2372	
1D	Short access Oxy/Sal vs. Oxy/093 rewards	Mixed model	Day F(2,34) = 3.3337, *p* = 0.0476; Treatment F(1,17) = 0.3396, *p* = 0.5677; Day*treatment F(2,34) = 0.1049, *p* = 0.9007	
	Long access Oxy/Sal vs. Oxy/093 rewards	Mixed model	Day F(9,153) = 1.0092, *p* = 0.4352; Treatment F(1,17) = 1.3997, *p* = 0.2530; Day*Treatment F(9,153) = 0.6683, *p* = 0.7236	
1E	Extinction (avg. last 3 days) vs. reinstatement and saline vs. 093 active lever presses	Mixed model	Session F(1,17) = 15.8118, *p* = 0.0010; Treatment F(1,17) = 0.2578, *p* = 0.6182; Session*treatment F(1,17) = 0.5216, *p* = 0.4800	
1F	Oxy/Sal vs. Oxy/093 cue‐induced reinstatement within session active lever presses	Mixed model	Time F(11,187) = 9.5040, *p *< 0.0001; Treatment F(1,17) = 0.3534, *p* = 0.5600; Time*treatment F(11,187) = 1.0446, *p* = 0.4090	
2D	Oxy/Sal vs. Oxy/093 NAc membrane GLT‐1/calnexin expression (% of saline)—middle band	Shapiro–Wilk	Oxy/Sal W = 0.8023, *p* = 0.0432; Oxy/093 W = 0.8698, *p* = 0.2655	*n* = 4 oxycodone/saline males *n* = 3 oxycodone/saline females *n* = 3 oxycodone/093 males *n* = 2 oxycodone/093 females
		Levene	F(1,10) = 1.0351; *p* = 0.3329	
		Wilcoxon exact test with Bonferroni correction (*α* = 0.0125)	*p* = 0.2652	
2E (Full)	Oxy/Sal vs. Oxy/093 NAc membrane GLT‐1/calnexin expression (% of saline)—full band	Shapiro–Wilk	Oxy/Sal W = 0.9575, *p* = 0.7974; Oxy/093 W = 0.8749, *p* = 0.2868	
		Levene	F(1,10) = 5.0334, *p* = 0.0487	
		Wilcoxon Exact Test with Bonferroni correction (*α* = 0.0125)	*p* = 0.4381	
2E (Top)	Oxy/Sal vs. Oxy/093 NAc membrane GLT‐1/calnexin expression (% of saline)—top band	Shapiro–Wilk	Oxy/Sal W = 0.9030, *p* = 0.3493; Oxy/093 W = 0.8745, *p* = 0.2851	
		Levene	F(1,10) = 9.8813; *p* = 0.0104	
		Wilcoxon exact test with Bonferroni correction (*α* = 0.0125)	*p* = 0.3194	
2E (Bottom)	Oxy/Sal vs. Oxy/093 NAc membrane GLT‐1/calnexin expression (% of saline)—bottom band	Shapiro–Wilk	Oxy/Sal W = 0.9391, *p* = 0.6310; Oxy/093 W = 0.9457, *p* = 0.7063
		Levene	F(1,10) = 0.0244, *p* = 0.8789
		Student's *t*‐test with Bonferroni correction (*α* = 0.0125)	t(10) = ‐1.7114, *p* = 0.1178
3A	Saline vs. cocaine CPP preference score	Mixed model	Test F(2,18) = 5.7684, *p* = 0.0116; Drug F(1,9) = 5.7886, *p* = 0.0395; Test*drug F(2,18) = 0.9242, *p* = 0.4149	*n* = 2 saline males *n* = 1 saline female *n* = 4 cocaine males *n* = 4 cocaine females
3C	Saline vs. cocaine NAc membrane GLT‐1/calnexin expression (% of saline)—middle band	Wilcoxon exact test with Bonferroni correction (*α* = 0.0125)	*p* = 0.0121	
3D (Full)	Saline vs. cocaine NAc membrane GLT‐1/calnexin expression (% of saline)—full band	Wilcoxon exact test with Bonferroni correction (*α* = 0.0125)	*p* = 0.4606	
3D (Top)	Saline vs. cocaine NAc membrane GLT‐1/calnexin expression (% of saline)—top band	Wilcoxon exact test with Bonferroni correction (*α* = 0.0125)	*p* = 0.0970	
3D (Bottom)	Saline vs. cocaine NAc memrane GLT‐1/calnexin expression (% of saline)—bottom band	Wilcoxon exact test with Bonferroni correction (*α* = 0.0125)	*p* = 0.2485	
4A	Saline vs. oxycodone CPP preference score	Mixed model	Test F(2,20) = 7.2256, *p* = 0.0043; Drug F(1,10) = 17.0817, *p* = 0.0020; Test*drug F(2,20) = 2.0610; *p* = 0.1435	*n* = 2 saline males *n* = 2 saline females *n* = 4 oxycodone males *n* = 4 oxycodone females
4C	Saline vs. Oxycodone NAc membrane GLT‐1/calnexin expression (% of saline)—middle band	Wilcoxon exact test with Bonferroni correction (*α* = 0.0125)	*p* = 0.4667	
4D (Full)	Saline vs. Oxycodone NAc membrane GLT‐1/calnexin expression (% of saline)—full band	Wilcoxon exact rest with Bonferroni correction (*α* = 0.0125)	*p* = 0.0364	
4D (Top)	Saline vs. Oxycodone NAc membrane GLT‐1/calnexin expression (% of saline)—top band	Wilcoxon exact test with Bonferroni correction (*α* = 0.0125)	*p* = 0.5697	
4D (Bottom)	Saline vs. Oxycodone NAc membrane GLT‐1/calnexin expression (% of saline)—bottom band	Wilcoxon exact test with Bonferroni correction (*α* = 0.0125)	*p* = 0.0242	
S1A	Short access active vs. inactive lever (by sex)	Mixed model	Sex F(1,17) = 1.253, *p* = 0.2785; Day (2,68) = 2.1730, *p* = 0.1217; Lever F(1,17) = 9.9534, *p* = 0.0058; Sex*day F(2,68) = 0.9064, *p* = 0.4088; Sex*lever F(1,17) = 0.5794, *p* = 0.4570; Day*lever F(2,68) = 1.6219, *p* = 0.2051, Sex*day*lever F(2,68) = 0.4033, *p* = 0.6697	*n* = 4 oxycodone/saline males *n* = 5 oxycodone/saline females *n* = 6 oxycodone/093 males *n* = 4 oxycodone/093 females
	Long access active vs. inactive lever (by sex)	Mixed model	Sex F(1,17) = 0.3553, *p* = 0.5590; Day F(9,306) = 1.2225, *p* = 0.2804; Lever F(1,17) = 52.3839, *p *< 0.0001; Sex*Day F(9,306) = 2.7405, *p* = 0.0043; Sex*lever F(1,17) = 0.0015, *p* = 0.9697; Day*lever F(9,306) = 0.8630, *p* = 0.5588; Sex*day*lever F(9,306) = 0.9252, *p* = 0.5033	
	Long access active vs. inactive lever (by sex)	Post‐hoc Tukey HSD all pairwise comparisons	Sex*Day: Female Day 7 vs. Female Day 12 *p* = 0.0134; Female Day 8 vs. Female Day 12 *p* = 0.0438; no other statistically significant differences	
	Extinction active vs. inactive lever (by sex)	Mixed model	Sex F(1,17) = 0.5050, *p* = 0.4869; Day F(3,102) = 1.7151, *p* = 0.1686; Lever F(1,17) = 3.9892, *p* = 0.0621; Sex*day F(3,102) = 3.6766, *p* = 0.0146; Sex*lever F(1,17) = 2.3082, *p* = 0.1471; Day*lever F(3,102) = 0.0436, *p* = 0.9878; Sex*day*lever F(3,102) = 0.3262, *p* = 0.8064	
	Extinction active vs. inactive lever (by sex)	Post‐hoc Tukey HSD All Pairwise Comparisons	Sex*Day: No statistically significant differences	
	Extinction + treatment active vs. inactive lever (by sex)	Mixed model	Sex F(1,17) = 0.0759, *p* = 0.7863; Day F(5,170) = 1.4839, *p* = 0.1975; Lever F(1,17) = 0.1008, *p* = 0.7548; Sex*Day F(5,170) = 0.9967, *p* = 0.4214; Sex*lever F(1,17) = 0.5231, *p* = 0.4794; Day*lever F(5,170) = 0.5948, *p* = 0.7039; Sex*day*lever F(5,170) = 2.2011, *p* = 0.0564	
S1D	Short access Oxy/Sal vs. Oxy/093 active lever presses (by sex)	Mixed model	Sex F(1,15) = 1.0835, *p* = 0.3144; Day F(2,30) = 2.4973, *p* = 0.0993; Treatment F(1,15) = 0.0001, *p* = 0.9925; Sex*Day F(2,30) = 0.5923, *p* = 0.5594; Sex*treatment F(1,15) = 0.1285, *p* = 0.7250; Treatment*day F(2,30) = 0.2027, *p* = 0.8176; Sex*treatment*day F(2,30) = 4.9709, *p* = 0.0137	
	Short access Oxy/Sal vs. Oxy/093 active lever presses (by sex)	Post‐hoc Tukey HSD All pairwise comparisons	Sex*treatment*day: No statistically significant differences	
	Long access Oxy/Sal vs. Oxy/093 active lever presses (by sex)	Mixed model	Sex F(1,15) = 0.2361, *p* = 0.6341; Day F(9,135) = 1.1928, *p* = 0.3046; Treatment F(1,15) = 1.4258, *p* = 0.2510; Sex*Day F(9,135) = 2.0080, *p* = 0.0429; Sex*treatment F(1,15) = 0.4750, *p* = 0.5012; Treatment*day F(9,135) = 0.8479, *p* = 0.5735; Sex*treatment*day F(9,135) = 1.6434, *p* = 0.1090	
	Long access Oxy/Sal vs. Oxy/093 active lever presses (by sex)	Post‐hoc Tukey HSD all pairwise comparisons	Sex*Day: No statistically significant differences	
	Extinction Oxy/Sal vs. Oxy/093 active lever presses (by sex)	Mixed model	Sex F(1,15) = 1.8161, *p* = 0.1978; Day F(3,45) = 0.8570, *p* = 0.4703; Treatment F(1,15) = 0.7376, *p* = 0.4040; Sex*Day F(3,45) = 2.1243, *p* = 0.1104; Sex*Treatment F(1,15) = 0.0524, *p* = 0.8220; Treatment*day F(3,45) = 0.1236, *p* = 0.9457; Sex*treatment*day F(3,45) = 0.0436, *p* = 0.9877	
	Extinction + treatment Oxy/Sal vs. Oxy/093 active lever presses (by sex)	Mixed model	Sex F(1,15) = 0.7459, *p* = 0.4014; Day F(5,75) = 3.1650, *p* = 0.0120; Treatment F(1,15) = 0.6363, *p* = 0.4375; Sex*Day F(5,75) = 2.1675, *p* = 0.0666; Sex*treatment F(1,15) = 0.7239, *p* = 0.4082; Treatment*day F(5,75) = 1.7294, *p* = 0.1382; Sex*treatment*day F(5,75) = 1.1457, *p* = 0.3440	
	Extinction + treatment Oxy/Sal vs. Oxy/093 active lever presses (by sex)	Post‐hoc Tukey HSD all pairwise comparisons	Day: Day 20 vs. Day 23 *p* = 0.0230; no other statistically significant differences	
S1E	Short access Oxy/Sal vs. Oxy/093 rewards (by sex)	Mixed model	Sex F(1,15) = 0.6628, *p* = 0.4283; Day F(2,30) = 3.5913, *p* = 0.0400; Treatment F(1,15) = 0.2216, *p* = 0.6446; Sex*Day F(2,30) = 0.4265, *p* = 0.6567; Sex*treatment F(1,15) = 1.1984; *p* = 0.2909; Treatment*day F(2,30) = 0.0458, *p* = 0.9553; Sex*treatment*day F(2,30) = 5.5441, *p* = 0.0089	
	Short access Oxy/Sal vs. Oxy/093 rewards (by sex)	Post‐hoc Tukey HSD all pairwise comparisons	Day: Day 1 vs. Day 3 *p* = 0.0499; no other statistically significant differences. Sex*treatment*day: no statistically significant differences	
	Long access Oxy/Sal vs. Oxy/093 rewards (by sex)	Mixed model	Sex F(1,15) = 0.8542, *p* = 0.3700; Day F(9,135) = 1.2935, *p* = 0.2460; Treatment F(1,15) = 1.3853, *p* = 0.2575; Sex*Day F(9,135) = 1.7498, *p* = 0.0836; Sex*treatment F(1,15) = 0.1121, *p* = 0.7424; Treatment*day F(9,135) = 0.9637, *p* = 0.4729; Sex*treatment*day F(9,135) = 1.6213, *p* = 0.1151	
S1F	Extinction (avg. last 3 days) vs. reinstatement and saline vs. 093 active lever presses (by sex)	Mixed model	Sex F(1,15) = 0.9859, *p* = 0.3365; Session F(1,15) = 14.5103, *p* = 0.0017; Treatment F(1,15) = 0.2624, *p* = 0.6159; Sex*session F(1,15) = 0.2744, *p* = 0.6081; Sex*treatment F(1,15) = 1.0972, *p* = 0.3115; Treatment*session F(1,15) = 0.4923, *p* = 0.4936; Sex*treatment*session F(1,15) = 0.4532, *p* = 0.5110	
S1G	Oxy/Sal vs. Oxy/093 cue‐induced reinstatement within session active lever presses (by sex)	Mixed model	Sex F(1,15) = 0.7800, *p* = 0.3911; Time F(11,165) = 9.1274, *p *< 0.0001; Treatment F(1,15) = 0.3533, *p* = 0.5611; Sex*time F(11,165) = 0.4160, *p* = 0.9477; Sex*treatment F(1,15) = 0.9418, *p* = 0.3472; Treatment*time F(11,165) = 1.0349, *p* = 0.4181; Sex*treatment*time F(11,165) = 1.1331, *p* = 0.3387	
S3A	093 CPP preference score	Shapiro–Wilk	Pre W = 0.9287, *p* = 0.3667; Pref W = 0.9409, *p* = 0.5099	*n* = 6 093 males *n* = 6 093 females
		Levene	F(1,22) = 0.4396, *p* = 0.5142	
		Paired Student's *t*‐test	t(11) = ‐1.3633, *p* = 0.2000	
S3B	Saline vs. 093 day distance traveled	Mixed model	Pairing F(3,66) = 10.4103, *p *< 0.0001; Drug F(1,22) = 30.2859, *p *< 0.0001; Pairing*Drug F(3,66) = 1.3779; *p* = 0.2572	
S3C (P1)	Pairing 1 saline vs. 093 within session distance traveled	Mixed model	Minute F(29,638) = 7.0186, *p *< 0.0001; Treatment F(1,22) = 30.7953, *p *< 0.0001; Minute*Treatment F(29,638) = 2.3814; *p *< 0.0001	
S3C (P3)	Pairing 3 saline vs. 093 within session distance traveled	Mixed model	Minute F(29,638) = 5.5986, *p *< 0.0001; Treatment F(1,22) = 11.6287, *p* = 0.0025; Minute*treatment F(29,638) = 2.1852; *p* = 0.0004	

## 3 Results

### 3.1 Treatment With 093 During Extinction of Oxycodone IVSA Does Not Reduce Cue‐Induced Reinstatement

**FIGURE 1:**
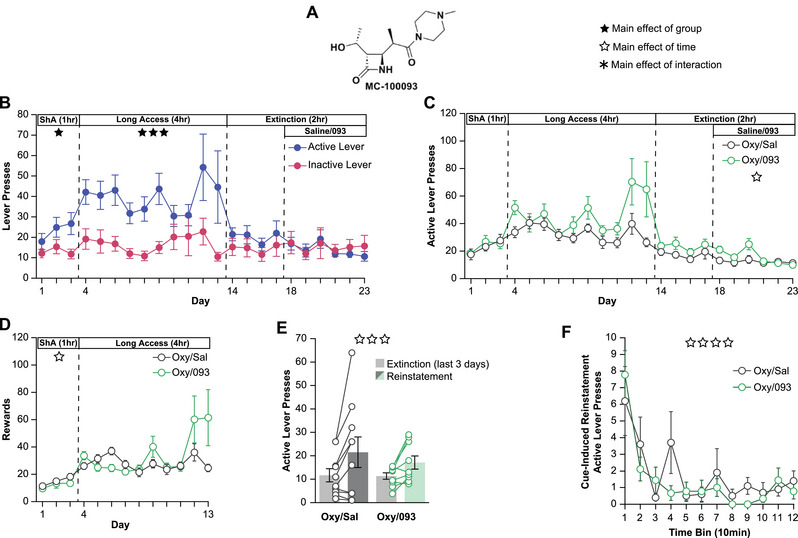
**093 does not reduce reinstatement to oxycodone self‐administration**. (**A)** Chemical structure of the beta lactam derivative MC‐100093. (**B)** Comparison of active lever (blue) and inactive lever (red) pressing across the entire cohort of Long Evans rats undergoing oxycodone (0.01 mg/kg/infusion) self‐administration shows animals learn to discriminate between active and inactive levers during short access (ShA), maintain active lever pressing during long access, reduce lever pressing during extinction, and that treatment with 093 or saline does not alter extinction behavior. (**C)** Active lever pressing between the saline (black) and 093 (green) groups is not different during any point of the experimental paradigm. (**D)** Similarly, there are no intrinsic differences in rewards earned between the animals that eventually received saline (grey) or 093 (green). (**E)** Treatment with 50 mg/kg 093 after the last six extinction sessions does not reduce subsequent cue‐induced reinstatement compared to the saline group. Light grey bars indicate the average active lever pressing during the last 3 days of extinction while the dark grey (saline) and light green (093) bars show active lever pressing during cue‐induced reinstatement. (**F)** Treatment with 093 did not alter active‐lever pressing behavior within the cue‐induced reinstatement session. Line graphs depict averages ± SEM. Bar graphs show averages ± SEM with points showing individual rats (*n* = 19, ten males and nine females split into ten saline and nine 093). Empty stars indicate significant main effect of time (either Day, Session, or Minute depending on the panel), filled stars a significant main effect of group, and asterisks a significant main effect of the interaction (n.s. = no significance; * *p < 0.05*; ** *p < 0.01*; *** *p < 0.001*; **** *p < 0.0001*).

To determine if 093 (Figure 1A) attenuates drug‐seeking for oxycodone, rats underwent intravenous self‐administration of 0.01 mg/kg oxycodone per infusion on an FR1 schedule. During the first three days of short access (ShA, 1 h/day), rats learned to discriminate between the active and inactive levers (Figure 1B, ShA). Rats transitioned to long access (LgA, 4 h/day) for ten days. During this time, they maintained their lever discrimination by engaging with the active lever significantly more than the inactive lever (Figure 1B, LgA). Upon initiation of extinction sessions (Ext, 4 days, 2 h/day), active lever pressing immediately dropped and the animals no longer discriminated between the active and inactive lever (Figure 1B, Ext). Rats received 50 mg/kg of 093 i.p., or saline, at the end of each of the last six extinction sessions (Figure 1B, Ext+Rx, 6 days, 2 h/day). We anecdotally observed acute hind limb motor deficits for approximately 10–15 min following i.p. injection of 093 in the absence of overt pain. No differences were observed in active lever presses between the oxycodone and saline (Oxy/Sal) or oxycodone and 093 (Oxy/093) groups throughout self‐administration (Figure 1C; ShA, LgA). In addition, no differences between the two groups were observed during the extinction sessions (Figure 1C; Ext). Consistent with previous findings with cocaine, 093 treatment did not alter extinction behavior compared to saline but both groups continued to reduce their active lever pressing over time (Figure 1C; Ext+Rx) (Knackstedt et al. 2021). Total rewards per session were recorded and no differences were seen between the Oxy/Sal and Oxy/093 groups during ShA (Figure 1D; ShA) or LgA (Figure 1D; LgA) sessions.

Twenty‐four hours after the last extinction session, rats went through a 2‐h cue‐induced reinstatement period. During this session, both groups reinstated self‐administration behavior by increasing their active lever presses (Figure 1E). However, there was no effect of treatment or the treatment*session interaction. We hypothesized that differences in active lever pressing patterns within the session could be present even in the absence of differences in total magnitude. We looked at active lever pressing during the cue‐induced reinstatement session in 10‐min bins and found no differences between the Oxy/Sal and Oxy/093 groups (Figure 1F).

Lastly, we analyzed our oxycodone self‐administration data considering sex as an additional variable (Figure S1). When comparing the active and inactive lever pressing by sex during the short access sessions, mixed model analyses revealed a significant main effect of the lever, but no main effect of sex, day, or any of the interactions (Figure S1A; ShA). Over the course of the long access sessions, alongside a significant main effect of Lever, there was a significant effect of the Sex*Day interaction. Post‐hoc Tukey pairwise comparisons identified differences between Female Day 7 and Female Day 12 as well as Female Day 8 and Female Day 12 (Figure S1A; LgA). These differences were driven by a single female animal drastically increasing their lever pressing during the final days of long access (Figure S1B,C). A similar Sex*Day interaction was found during the first stage of extinction, with no significant post‐hoc pairwise comparisons (Figure S1A; Ext). Similarly, when comparing the saline and 093 groups by sex, there was a significant Sex*Day interaction during long access but no significant post‐hoc pairwise comparisons (Supp.1D; LgA). Again, these findings were most likely driven by the single female animal highly engaging with the levers towards the end of long access (Figure S1B,C). A significant Sex*Day*Treatment interaction was also identified when comparing active lever presses and rewards earned by sex and treatment during short access, but no significant pairwise comparisons (Figure S1D,E; ShA). Finally, when evaluating reinstatement by treatment and sex, there was a significant main effect of session, indicating reinstatement, but no main effect of sex, treatment, or any of the interactions (Figure S1F). Taken together, these results suggest the sex of the rats was unlikely to be playing a substantial role in our self‐administration study.

### 3.2 Treatment With 093 During Extinction to Oxycodone IVSA Does Not Increase Membrane GLT‐1 Expression in the NAc

**FIGURE 2:**
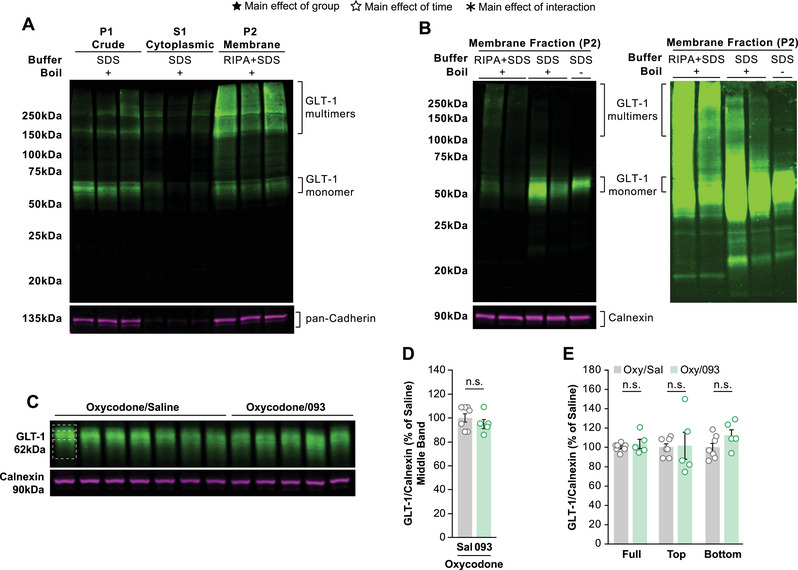
**093 does not positively modulate NAc membrane GLT‐1 expression following oxycodone self‐administration**. (**A)** Western blot of fractionated rat bulk forebrain tissue for protocol validation probed for GLT‐1 and the membrane protein pan‐cadherin. Every third lane is the same animal, and every sample was heated to 95°C before loading. Lanes 1–3 are the crude fraction reconstituted in homogenization buffer + SDS (HB+SDS), lanes 4–6 are the cytoplasmic fraction reconstituted with HB+SDS, and lanes 7–9 are the membrane fraction reconstituted with RIPA buffer + SDS. Protein separated by the fractionation protocol segregates between cytoplasmic and membrane compartments. Higher molecular weight signal is present on the GLT‐1 channel. (**B)** Western blot of membrane fraction from rat bulk forebrain tissue to test the roles of reconstitution buffer and sample heating before loading in producing higher molecular weight signal when probing for GLT‐1. During the fractionation process each sample was split in half before the final reconstitution to produce the membrane fraction. One set were reconstituted with RIPA+SDS and the other set with HB+SDS. Lanes 1 and 2 recapitulate the higher molecular weight GLT‐1 signal when reconstituting with RIPA+SDS. Lanes 3–4 show a reduction in this higher molecular weight signal when reconstituting without RIPA buffer. In lane 5 when the sample is additionally not heated before loading almost no signal is observed outside of the expected 62 kDa band. (**C)** Twenty‐four hours after cue‐induced reinstatement nucleus accumbens (NAc) tissue was collected and fractionated. Membrane fractions were probed for GLT‐1 and the membrane protein calnexin. (**D)** Quantification of the middle GLT‐1 band reveals no differences between the Oxy/Sal (grey) and Oxy/093 (green) groups. (**E)** Quantification of the full band, top band, and bottom band show no differences between the two groups. Bars depict average ± SEM and points show individual rats. Empty stars indicate significant main effect of time, filled stars a significant main effect of group, and asterisks a significant main effect of the interaction (*n* = 12; seven males and five females split into seven Oxy/Sal and five Oxy/093; n.s. = no significance; * *p < 0.05*; ** *p < 0.01*; *** *p < 0.001*; **** *p < 0.0001*).

Beta‐lactams are thought to reduce drug‐seeking by increasing membrane expression of the astrocytic glutamate transporter GLT‐1 within the NAc (Knackstedt et al. 2010, 2021). Because 093 did not reduce cue‐induced reinstatement of oxycodone self‐administration, we hypothesized that 093 failed to upregulate membrane GLT‐1 expression. To test this, we performed western blot experiments on NAc tissue from the rats that underwent oxycodone self‐administration using a fractionation protocol often used in the study of membrane GLT‐1 (Knackstedt et al. 2010; Knackstedt et al. 2021; Sari et al. 2024; Shen et al. 2014; Sepulveda‐Orengo et al. 2018). We first sought to validate the protocol in our own hands and ran western blots of the P1 (crude), S1 (cytoplasmic), and P2 (membrane) fractions to confirm the separation of cytoplasmic and membrane proteins (Figure 2A, Supp.2A). Both GLT‐1 and pan‐cadherin, a membrane marker, were observed in P1 (lanes 1–3). However, the protein that was successfully fractionated out from P1 was able to segregate between cytoplasmic (lanes 4–6) and membrane (lanes 7–9) fractions as only small amounts of GLT‐1 and almost no pan‐cadherin were observed in S1.

In addition, we detected the presence of higher molecular weight bands of GLT‐1 (Figure 2A; Figure S2A). We identified the reconstitution of the membrane fraction with RIPA buffer as a potential factor in this oligomerization. When membrane fractions were instead reconstituted in the absence of RIPA buffer, we observed a reduction in this higher molecular weight signal (Figure 2B; Figure S2B lanes 3–4). When samples reconstituted without RIPA buffer were additionally not boiled before loading, almost no signal was detected above the expected 62 kDa molecular weight of the GLT‐1 monomer (Figure 2B lane 5). As such, all subsequent experiments examining membrane GLT‐1 were performed in the absence of RIPA buffer and without boiling the lysates before loading the western blot.

We next tested the hypothesis that 093 treatment during extinction did not alter cue‐induced reinstatement to oxycodone self‐administration because it did not upregulate membrane expression of GLT‐1 in the NAc. Membrane fractions of NAc tissue from this cohort were examined by western blot (Figure 2C; Supp.2C). Our modified fractionation and loading protocol reproduced the removal of higher molecular weight GLT‐1 bands. We identified a prominent central GLT‐1 band at 62 kDa alongside a softened signal above and below this central band. GLT‐1 is known to undergo significant post‐translational modifications (PTMs) which could be reflected in these additional smeared signals around the middle band (Pajarillo et al. 2019; Peterson and Binder 2019). Accordingly, we quantified this central band, the upper and lower “bands”, as well as the full signal. Measuring just the middle band revealed no differences in NAc membrane GLT‐1 expression between the Oxy/Sal and Oxy/093 groups following cue‐induced reinstatement (Figure 2D). Similarly, there were no differences in the top, middle, or full band quantifications (Figure 2E). Due to small group sizes when separating groups by sex and treatment, sex was not considered as a factor during statistical testing.

### 3.3 Membrane GLT‐1 Expression Is Decreased Following CPP for Cocaine

Interestingly, previous reports have shown that beta‐lactams such as ceftriaxone do not modulate membrane GLT‐1 expression within the NAc of naïve animals (Knackstedt et al. 2010; Smaga et al. 2020). In other words, beta‐lactams appear to require an exposure‐dependent decrease in GLT‐1, such as cocaine, to influence its expression. It has been consistently shown that repeated exposure to cocaine, both self‐administered and experimenter‐delivered, reduces membrane expression of GLT‐1 in the NAc (Knackstedt et al. 2010; Smaga et al. 2020, Niedzielska‐Andres et al. 2019). However, this has yet to be shown for oxycodone. We hypothesized that 093's inability to positively modulate GLT‐1's membrane expression in our IVSA experiment was because oxycodone alone did not create the exposure‐dependent decrease needed for beta‐lactams to have an effect.

**FIGURE 3:**
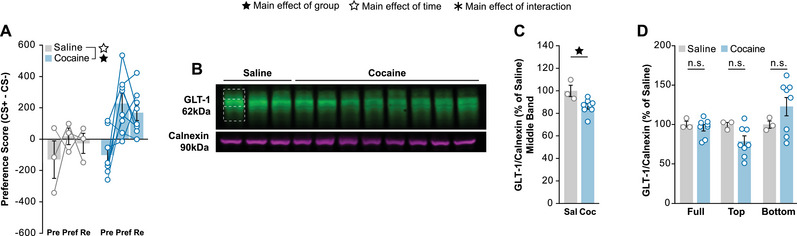
**Cocaine reduces NAc membrane GLT‐1 expression. (A)** Conditioned place preference test for cocaine in C57BL/6J mice. Animals went through two‐days of baseline pretest, eight days of conditioning alternating between the conditioned and unconditioned side, a preference test, seven days of forced home cage abstinence, and a final retest. Cocaine produces a preference for the stimulus paired side compared to saline. (**B)** Western blot of NAc membrane fractions probing for GLT‐1 and calnexin comparing saline and cocaine. (**C)** Repeated acute exposure to cocaine reduces NAc membrane GLT‐1 expression in the middle band compared to saline while (**D)** no differences are seen in the full, top, or bottom bands. Bars represent average ± SEM and points show individual mice. Empty stars indicate significant main effect of time, filled stars a significant main effect of group, and asterisks a significant main effect of the interaction (*n* = 11 with 6 males and 5 females split into 3 saline and 8 cocaine; n.s. = no significance; * *p < 0.05*; ** *p < 0.01*; *** *p < 0.001*; **** *p < 0.0001*).

We first looked to confirm that our modified western blot protocol would be able to detect differences in GLT‐1 expression. To do so, we ran CPP in mice for 15 mg/kg cocaine using a protocol identical to ones previously used to detect differences in membrane GLT‐1 expression by cocaine (Niedzielska‐Andres et al. 2019). As expected, cocaine‐treated mice exhibited CPP to the drug‐injection side (Figure 3A). NAc tissue was collected, and the membrane fraction was examined by western blot (Figure 3B; Supp.2D). When quantifying only the middle band of the GLT‐1 signal we found a slight but significant decrease in membrane expression within the NAc of cocaine‐treated animals compare to saline‐treated (Figure 3C). This difference is of comparable magnitude to previous reports using this cocaine CPP paradigm as well as in IVSA for cocaine (Knackstedt et al. 2010; Niedzielska‐Andres et al. 2019, Knackstedt et al. 2021). The full, top, and bottom bands showed no differences between the saline and cocaine‐treated animals (Figure 3D). Sex was not considered as a factor due to small group sizes when separating groups by treatment and sex.

### 3.4 Membrane GLT‐1 Expression Is Not Decreased Following CPP for Oxycodone

**FIGURE 4:**
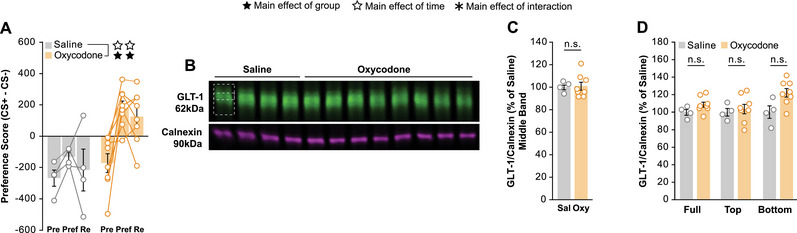
**Oxycodone does not reduce NAc membrane GLT‐1 expression. (A)** Using the same paradigm as Figure 3, animals show a preference for the side conditioned to oxycodone while saline treated animals do not show a preference for the conditioned side. (**B)** NAc membrane tissue examined by western blot and probed for GLT‐1 and calnexin. (**C)** Quantification showed that repeated exposure to oxycodone did not reduce NAc GLT‐1 membrane expression in the middle band. (**D)** Examination of the top band revealed no differences between saline and oxycodone while the full and bottom band trend towards an increase in expression in the oxycodone treated animals. Bars represent average ± SEM and points show individual mice. Empty stars indicate significant main effect of time, filled stars a significant main effect of group, and asterisks a significant main effect of the interaction (*n* = 12 with 6 males and 6 females split into 4 saline and 8 cocaine; n.s. = no significance; * *p < 0.05*; ** *p < 0.01*; *** *p < 0.001*; **** *p < 0.0001*).

After confirming our CPP paradigm and western blot protocol were sensitive to changes in membrane GLT‐1 expression, we proceeded to test our hypothesis. To examine whether oxycodone alone modulates membrane GLT‐1 in the NAc, we ran the same CPP experiment outlined above but with oxycodone. Similar to cocaine, oxycodone produced a strong preference for the conditioned side that saline did not (Figure 4A), and membrane fractions of NAc tissue were subsequently probed for GLT‐1 by western blot (Figure 4B; Supp.2E). Quantification revealed no differences between the saline and oxycodone treated animals in the middle GLT‐1 band (Figure 4C). In addition, we detected no differences in the top band (Figure 4D). Interestingly, we did find a trend for an increase in GLT‐1 in the full and bottom band of oxycodone‐treated animals compared to saline‐treated (Figure 4D).

We ran a final CPP for 50 mg/kg 093 alone to test whether the beta‐lactam intrinsically produced a preference or aversion. The acute motor deficit produced by 093 in the rats was recapitulated in these mice. Despite this, the mice did not develop a preference or aversion to the side conditioned to 093 (Supp.3A). The locomotor activity during the conditioning days was recorded and revealed that the animals traveled significantly less on days they received 093 compared to days they got saline (Supp.3B). We also sought to characterize the temporal aspect of this deficit and investigated the within‐session locomotion for the first (P1) and third (P3) 093/saline pairings. When examining 1‐min bins, on days the animals received 093 they traveled significantly less for the first 15 min before renormalizing their activity to the saline‐treated days (Supp.3C). Separating groups by treatment and sex resulted in group sizes too small to consider sex as a factor.

## 4 Discussion

The present study investigated the novel beta‐lactam derivative MC‐100093's efficacy in reducing cue‐induced reinstatement to oxycodone self‐administration. Our intravenous self‐administration cohort unexpectedly revealed that 093 was unable to attenuate drug seeking for oxycodone. Concomitantly 093 did not increase membrane expression of the glutamate transporter GLT‐1 in the NAc. Subsequent experiments suggest that the lack of effect of 093 on GLT‐1 expression and oxycodone‐seeking behavior in the IVSA study may be due to oxycodone alone not altering GLT‐1 expression. We unexpectedly observed acute motor deficits immediately following i.p. injection of 093 in both rats and mice, although this did not lead to an aversion or preference for the 093 paired side in CPP.

To determine whether 093 was capable of attenuating oxycodone relapse‐like behavior, we employed a self‐administration–extinction–reinstatement model of drug seeking, similar to the previously used to investigate 093 and cocaine (Knackstedt et al. 2021). Rats quickly learned to intravenously self‐administer oxycodone and discriminate between the active and inactive levers. During extinction sessions, rats reduced their lever pressing and no longer differentiated between the two levers. Treatment with 093 did not alter extinction behavior and the Oxy/Sal and Oxy/093 groups had statistically similar active lever pressing and earned rewards. In addition, treatment with 093 failed to attenuate reinstatement. Our self‐administration study included both male and female rats in group sizes sufficiently large enough for the examination of sex as a variable. We identified no significant effect of sex. In certain cases, there was a significant effect of an interaction including sex, but no relevant post‐hoc comparisons were found. Most of these effects were primarily driven by a single high‐responding female during the end of long access. The lack of observed sex differences is consistent with previous work using IVSA models (Guha et al. 2022; Hinds et al. 2023). However, other groups have found sex differences during oxycodone self‐administration (Mavrikaki et al. 2017; Kimbrough et al. 2020; Fulenwider et al. 2019; Illenberger et al. 2023). Methodological differences in duration of sessions, number of self‐administration days, route of administration, oxycodone dosage, and whether or not that dosage was adjusted for the animal's body weight could account for these diverging behavioral results.

Following reinstatement, expression of GLT‐1 in the NAc was examined and no differences between the saline and 093 groups were found. This is in contrast with the original paper describing 093's ability to attenuate cocaine seeking (Knackstedt et al. 2021). One notable difference between our two studies is the exact time point of tissue collection. Knackstedt et al. used two separate cohorts, one that underwent extinction–reinstatement and the other solely extinction, and collected tissue for western blots from the latter group. Our tissue was collected in the same cohort that underwent reinstatement 24 h after the reinstatement session. It is possible that the reinstatement session itself produced an experience‐dependent change in GLT‐1 expression that masked differences we would have seen if we had collected tissue before reinstatement. However, this would not account for our CPP studies in which both the cocaine and oxycodone groups went through a reinstatement session, and within which the cocaine group still showed a decrease in GLT‐1 expression in the NAc that was absent in the oxycodone group.

Although, functionally active GLT‐1 is thought to be a multimeric protein, the non‐covalent bonds between the monomers are expected to dissociate in the presence of detergents, reducing agents, and boiling (Danbolt 2001; Yernool et al. 2004). When processing lysates to probe for GLT‐1 by western blot, certain conditions can cause the protein to artifactually multimerize (Danbolt 2001; Haugeto et al. 1996). Our western blot experiments recapitulate this previously described behavior of GLT‐1 ex vivo, which can confound quantification if focused specifically on the monomeric form. While in our hands, we found RIPA buffer to cause multimerization, the consistency of this phenomenon is unclear. Reports have successfully used RIPA buffer to solubilize fractions and only observe GLT‐1 in its monomeric form, while others show higher molecular weight species in these fractions (Moshrefi‐Ravasdjani et al. 2018; Khatri et al. 2024). Conversely, other studies report these higher molecular weight species even in the absence of RIPA buffer (although their homogenization buffer uses many of the same constituents present in RIPA buffer) (Genda et al. 2011). In addition, a previous study identified the need to immediately homogenize tissue with reducing agents and SDS after collection—without freezing for storage—to prevent multimerization (Haugeto et al. 1996). Given the variability across studies regarding the conditions that produce this higher molecular weight signal, our experiments underscore the importance of validating protocols and presenting full, uncropped western blots. This practice enables readers to accurately assess the complete migration patterns of the probed proteins. Our western blots also highlight that GLT‐1 expression alone may not dictate phenotypic outcomes. The prominent monomeric band surrounded by a fainter signal that we observe can be seen in previous studies using western blots to quantify changes in NAc GLT‐1 following cocaine self‐administration (Sepulveda‐Orengo et al. 2018). GLT‐1 is known to undergo a plethora of PTMs that could influence its function even in the absence of differences in expression, which may be reflected in this signal (Pajarillo et al. 2019; Peterson and Binder 2019). These PTMs include phosphorylation, glycosylation, ubiquitination, palmitoylation, nitrosylation, and sumoylation and could influence the function of GLT‐1 even in the absence of differences in protein expression. Future studies will be needed to explore how these PTMs change following exposure to drugs of abuse and their subsequent functional outcomes.

To elucidate the relationship between oxycodone and NAc membrane GLT‐1 expression, we used a CPP model. Our study found that oxycodone exposure alone does not reduce NAc membrane GLT‐1 expression. The CPP paradigm only features experimenter‐administered drug, while self‐administration is an operant task, raising the possibility that changes in GLT‐1 expression following oxycodone treatment require operant behavior, and our experiments do not directly test that. Nonetheless, for cocaine, these reductions are seen in both self‐administration and experimenter‐administered settings, although this does not mean the same findings would translate over to oxycodone or opioids in general (Knackstedt et al. 2010, 2021; Niedzielska‐Andres et al. 2019). It is also possible that GLT‐1 expression changes could be observed following oxycodone exposure using different dosages or delivery schedules not tested for in our CPP studies. Our CPP experiments also used mice as opposed to rats as in the original IVSA experiment. While differences may be present, both mice and rats have been used to study ceftriaxone (Knackstedt et al. 2010; Freet and Lawrence 2015). Our lab has previously established the utility of CPP for studying opioids and beta‐lactams in mice (Hearing et al. 2016). In addition, our cocaine CPP experiment recapitulates cocaine‐induced decreases in NAc membrane GLT‐1 expression seen in analogous studies done with rats using a near‐identical protocol (Niedzielska‐Andres et al. 2019).

Finally, we tested whether 093 alone was inherently rewarding or aversive. While we found that it was not, we did observe an acute locomotor deficit following i.p. injection of 093. This motor deficit began 3–5 min following the injection and lasted for around 15 min. The original 093 report also tested locomotion; however, this experiment was done 1 day after the most recent 093 injection and binned data in 10‐min intervals (Knackstedt et al. 2021). As our results suggest an acute effect lasting only 15 min immediately ensuing the injection it is unlikely their experiment would have captured this phenotype. Since then, another study examining 093 performed an open‐field test. While they report no differences in distance traveled, male animals appeared to statistically trend towards lower locomotion (León et al. 2023). In addition, they only measured the total distance traveled over a 30‐min period with no within‐session data and is unclear if there was a delay between the injection time and initiation of the open field test (as a battery of tests were performed on the same day). Interestingly, this report also investigated the efficacy of 093 in reducing ethanol seeking and found that in high ethanol preference mice, animals receiving treatment with 093 did not change their total ethanol intake but female mice slightly reduced their preference for ethanol. In addition, they did not see modulation of GLT‐1 expression by 093, but this was done in a separate cohort of animals and in the medial prefrontal cortex and hippocampus.

Studies on GLT‐1 expression in the NAc and opioids show mixed results. To our knowledge, only one other study has investigated GLT‐1 expression after oxycodone treatment in the context of an A‐B‐A‐B oxycodone/cocaine polysubstance intravenous self‐administration model (Khatri et al. 2024). They found reduced expression of GLT‐1 in the NAc core in their oxycodone/cocaine group compared to the food/cocaine group. However, with the polysubstance use model, it is difficult to determine if exposure to oxycodone alone was responsible for this decrease or the combinatorial use of oxycodone with cocaine. Like cocaine, intravenous self‐administration of heroin in rats reduces GLT‐1 expression in the NAc and impairs glutamate uptake (Shen et al. 2014). Repeated intraperitoneal (i.p.) treatment with morphine also reduces NAc GLT‐1 expression which is rescued following treatment with ceftriaxone or 093 (Sari et al. 2024). Non‐contingent treatment using subcutaneously implanted morphine pellets decreased GLT‐1 mRNA in the striatum (Ozawa et al. 2001). Pretreatment gene delivery of GLT‐1 in the NAc shell by adeno‐associated virus prevented CPP for morphine (Fujio et al. 2005). Noncontingent i.p. injection with fentanyl reduces NAc GLT‐1 expression that is rescued with 093 or ceftriaxone (Alasmari et al. 2024). Results from hydrocodone studies are diverging, with one finding hydrocodone exposure nor treatment with ceftriaxone having an effect on NAc GLT‐1 expression while two others showing a decrease in GLT‐1 expression and rescue with ceftriaxone (Alshehri et al. 2018; Wong and Sari 2023, 2024). Although, all of these reports come from the same group and provided the drug i.p., differences in hydrocodone dose, number of exposures, rat genotype (alcohol‐preferring “P rats” vs. wildtype), and presence or absence of a preceding behavioral test could all play a role in the differing results. It has been previously suggested that the δ‐opioid receptor (DOR) may play an important role in modulating GLT‐1 expression (Roberts‐Wolfe and Kalivas 2015). This idea was based on a report demonstrating the potential role of DOR in reducing GLT‐1 expression in cultured astrocytes (Thorlin et al. 1998) [but see otherwise (Liang et al. 2014)]. When comparing the opioids described in the literature that do affect GLT‐1 expression in vivo and those that do not, morphine (which is also a metabolite of heroin) and fentanyl have a magnitude stronger binding affinity for the DOR than oxycodone (Codd et al. 1995; Eshleman et al.^2020^). Our findings with oxycodone, of similar affinity to hydrocodone, lend further credence to the potential role of DOR in modulating GLT‐1 expression (Codd et al. 1995). Interestingly, a recent study within the amygdala has tied morphine abstinence‐dependent increases in κ‐opioid receptor (KOR) ligand expression upstream to changes in GLT‐1 expression (Zan et al. 2021). Future studies will be needed to further clarify in vivo the relationship between the differential activation of distinct opioid receptors by various exogenous opioids and the resulting changes in GLT‐1 expression alongside potential brain region differences.

Taken together, our results show that the novel beta‐lactam derivative MC‐100093 was unable to reduce cue‐induced reinstatement for oxycodone self‐administration. Our data highlight that these compounds may have drug‐specific differences in efficacy, even within the same class of drugs. Investigations into the upstream mechanisms that drive changes in GLT‐1 expression, as well as the functional consequences of differing PTMs following drug exposure, could further clarify how beta‐lactams could be best translated as a treatment option for OUD.

## Author Contributions

D.W.H., Y.A‐C., M.E., and M.J.T. conceived and designed the study. D.W.H., Y.A‐C., B.H., A.R., and M.A.B. conducted experiments. W.E.C. and M.A‐G. provided critical reagents. D.W.H. assembled the figures. D.W.H., Y.A‐C., M.E., and M.J.T wrote the manuscript. All authors revised and provided approval for the publication of this work.

## Conflicts of Interest

The authors declare no conflicts of interest.

## Peer Review

The peer review history for this article is available at https://publons.com/publon/10.1002/brb3.70616

## Supporting information




**Supplementary Figure 1. No effect of sex on oxycodone self‐administration, extinction, reinstatement, or treatment with 093. A)** Comparison of active and inactive lever pressing by sex. Male and female **B)** active and **C)** inactive lever pressing between days 7 and 13 highlight the spiking in lever pressing seen in the females is primarily driven by one female rat. Oxycodone/saline and oxycodone/093 **D)** active lever pressing and **E)** rewards earned separated by sex. **F)** Comparison of extinction and reinstatement session active lever pressing separated by treatment and sex. **G)** Reinstatement within‐session active lever pressing by treatment and sex. Bar graphs in **F** show averages ± SEM with points showing individual rats. Line graphs in **A, D, E**, and **G** show averages ± SEM while lines in **B** and **C** are individual rats (n = 19, ten males and nine females split into ten saline and nine 093). Empty stars indicate significant main effect of time (either Day, Session, or Minute depending on the panel), filled black stars a significant main effect of group, filled grey stars a significant effect of sex, and asterisks a significant main effect of an interaction (n.s. = no significance; * *p<0.05*; ** *p< 0.01*; *** *p<0.001*; **** *p<0.0001*).

**Supplementary Figure 2. Full Western blots**. Full, uncropped western blot from **A)** Figure 2A (western blot fractionation validation) probing for pan‐Cadherin and GLT‐1. Full, uncropped western blots for **B)** Figure 2B (GLT‐1 multimerization validation), **C)** Figure 2C (oxycodone IVSA with saline/093), **D)** Figure 3B (cocaine CPP), and **E)** Figure 4B (oxycodone CPP) were probed for GLT‐1 and calnexin.

**Supplementary Figure 3. 093 causes an acute locomotor deficit that does not produce a conditioned preference or aversion. A)** Conditioned place preference for 093 in C57BL/6J mice showed no inherent preference or aversion for the 093‐paired side. **B)** Animals moved less on days they received 093 (dark grey) compared to saline (light grey). **C)** Within session locomotion revealed an acute motor deficit reflected by a reduction in distance traveled that recovered within 15‐minutes on 093 days (dark grey) compared to saline days (light grey). Line graphs depict averages ± SEM. Bar graphs show averages ± SEM with points showing individual mice (n = 12, 6 males and 6 females). Empty stars indicate significant main effect of time, filled stars a significant main effect of group, and asterisks a significant main effect of the interaction (n.s. = no significance; * *p<0.05*; ** *p< 0.01*; *** *p<0.001*; **** *p<0.0001*).

## Data Availability

All data will be made available to reasonable requests which can be sent to the corresponding author.
