# Supplementary material for: Differential Modulation of Glutamate Transporter‐1 by Cocaine and Oxycodone and the Efficacy of MC‐100093 to Reduce Reinstatement of Self‐Administration
Source: Brain Behav. 2025 Jul 10;15(7):e70616. doi: 10.1002/brb3.70616 (PMC12241825; doi:10.1002/brb3.70616)

Supplementary Figure 1

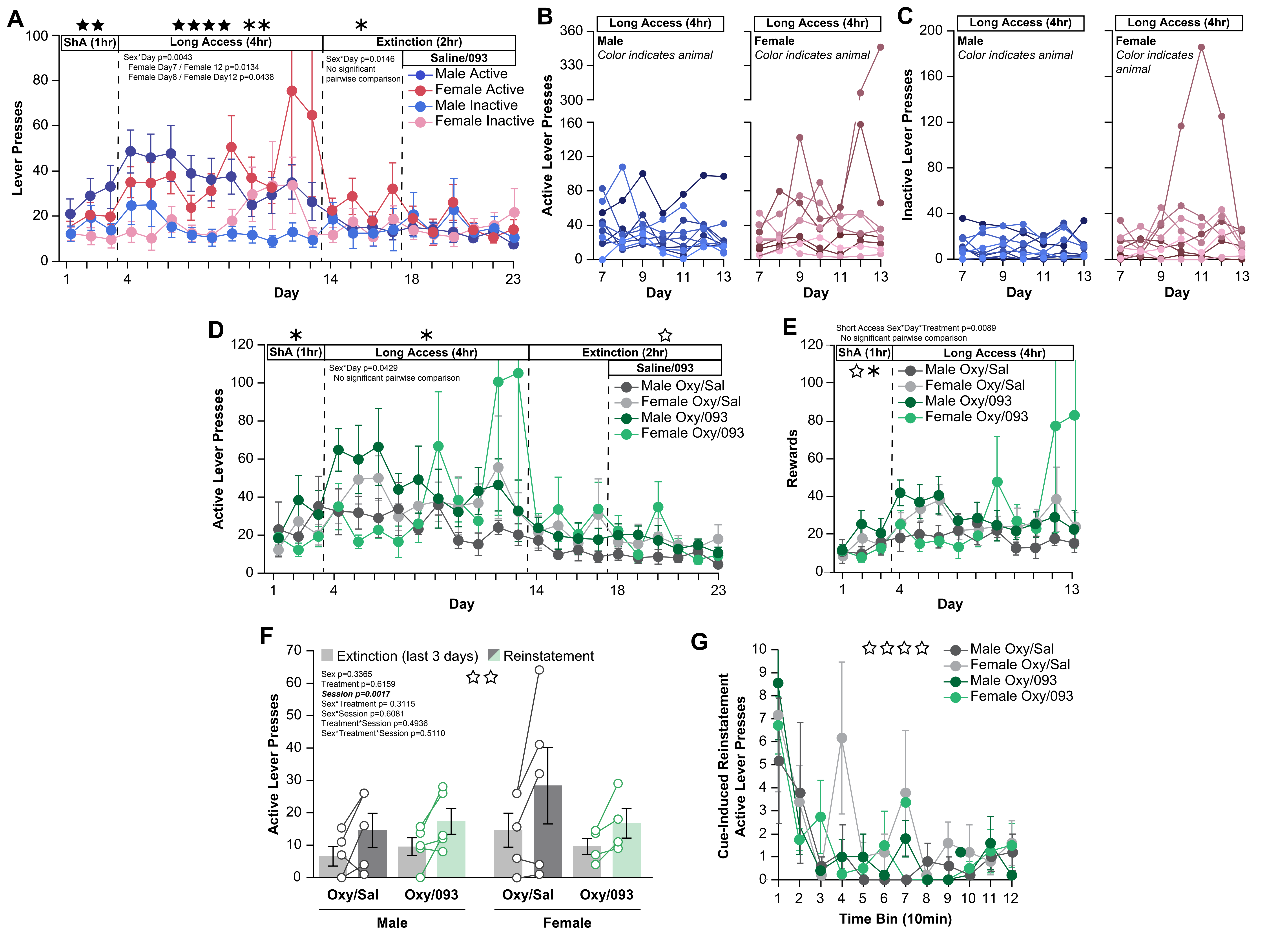

Supplement: Supplementary file 1 — Supplementary Figure 1. No effect of sex on oxycodone self‐administration, extinction, reinstatement, or treatment with 093. A) Comparison of active and inactive lever pressing by sex. Male and female B) active and C) inactive lever pressing between days 7 and 13 highlight the spiking in lever pressing seen in the females is primarily driven by one female rat. Oxycodone/saline and oxycodone/093 D) active lever pressing and E) rewards earned separated by sex. F) Comparison of extinction and reinstatement session active lever pressing separated by treatment and sex. G) Reinstatement within‐session active lever pressing by treatment and sex. Bar graphs in F show averages ± SEM with points showing individual rats. Line graphs in A, D, E, and G show averages ± SEM while lines in B and C are individual rats (n = 19, ten males and nine females split into ten saline and nine 093). Empty stars indicate significant main effect of time (either Day, Session, or Minute depending on the panel), filled black stars a significant main effect of group, filled grey stars a significant effect of sex, and asterisks a significant main effect of an interaction (n.s. = no significance; * p<0.05; ** p< 0.01; *** p<0.001; **** p<0.0001). [file BRB3-15-e70616-s003.pdf]
