# Supplementary figures and images for: Differential Modulation of Glutamate Transporter‐1 by Cocaine and Oxycodone and the Efficacy of MC‐100093 to Reduce Reinstatement of Self‐Administration
Source: Brain Behav. 2025 Jul 10;15(7):e70616. doi: 10.1002/brb3.70616 (PMC12241825; doi:10.1002/brb3.70616)

Supplementary Figure 2

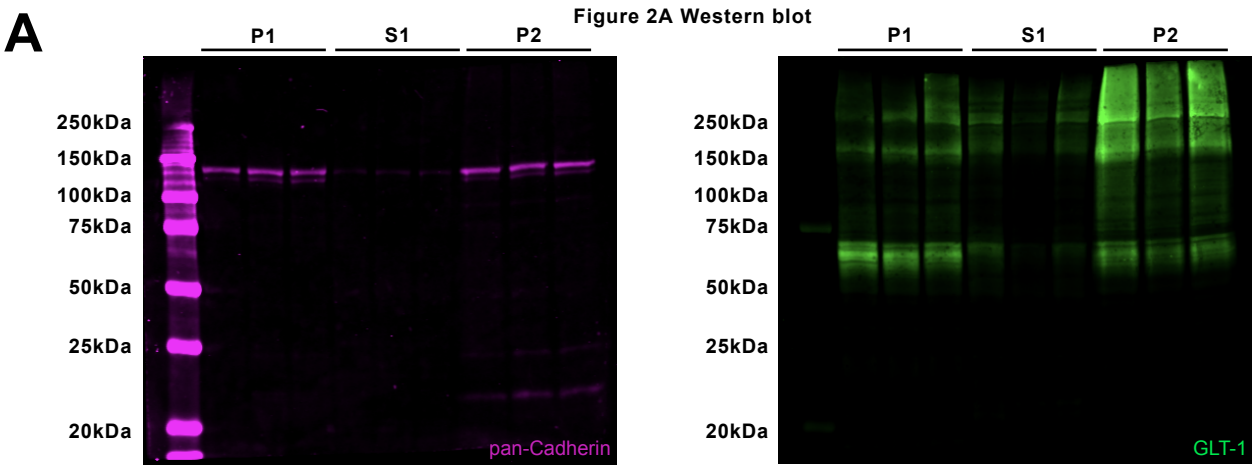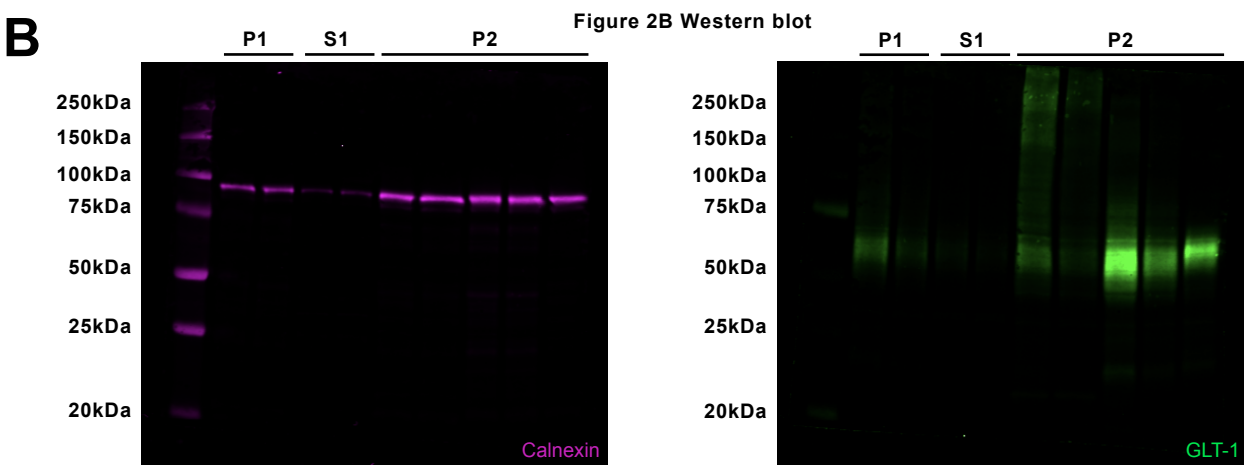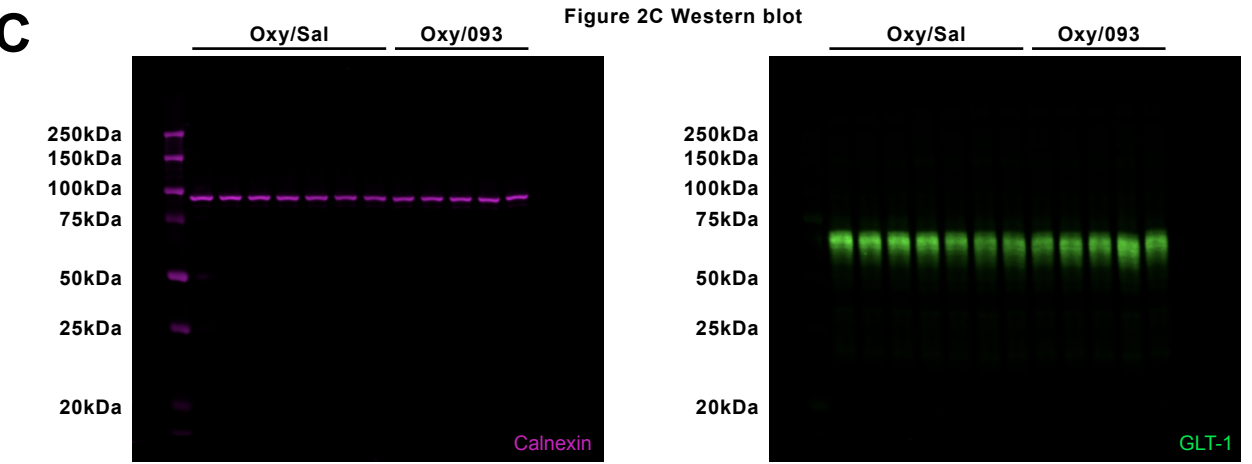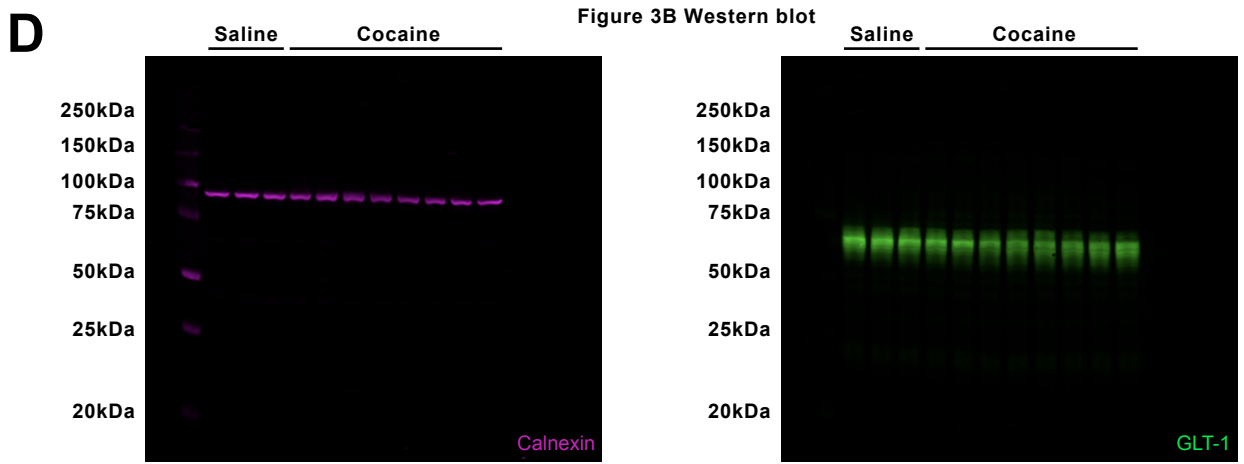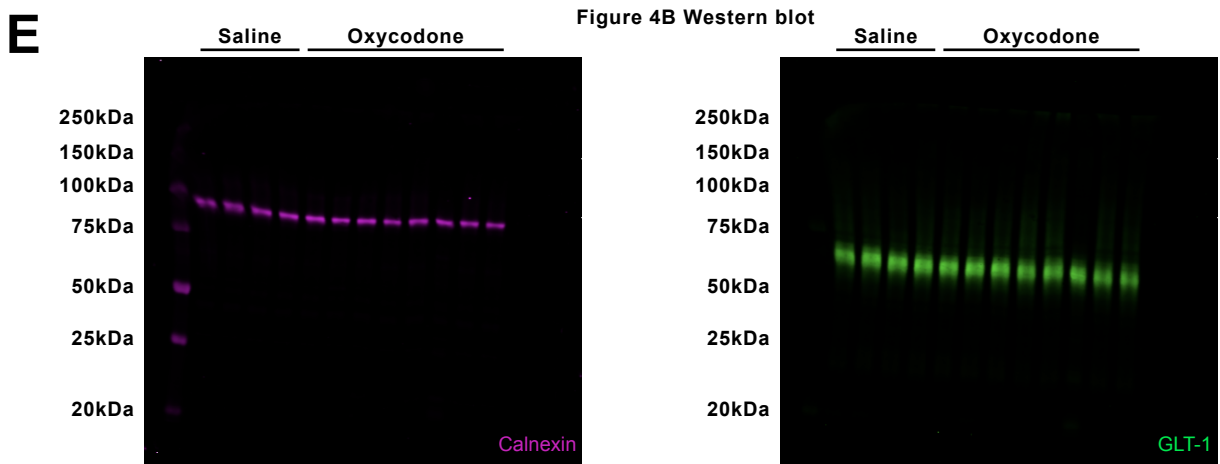

Supplement: Supplementary file 2 — Supplementary Figure 2. Full Western blots. Full, uncropped western blot from A) Figure 2A (western blot fractionation validation) probing for pan‐Cadherin and GLT‐1. Full, uncropped western blots for B) Figure 2B (GLT‐1 multimerization validation), C) Figure 2C (oxycodone IVSA with saline/093), D) Figure 3B (cocaine CPP), and E) Figure 4B (oxycodone CPP) were probed for GLT‐1 and calnexin. [file BRB3-15-e70616-s002.pdf]

Supplementary Figure 3

★ Main effect of group    ☆ Main effect of time    \* Main effect of interaction

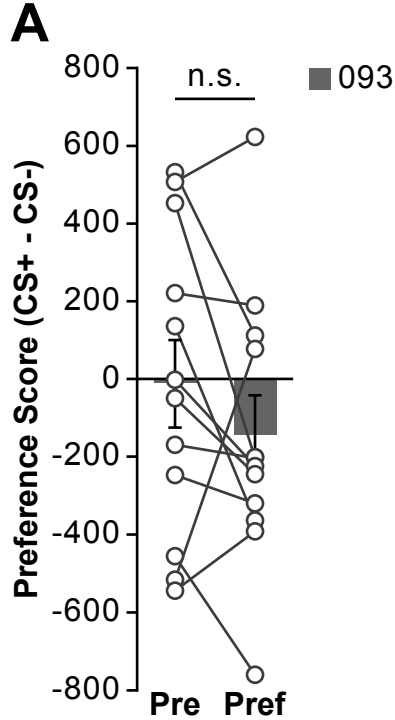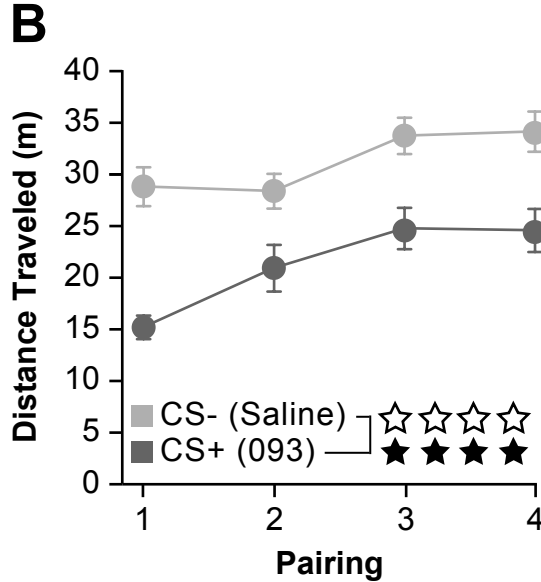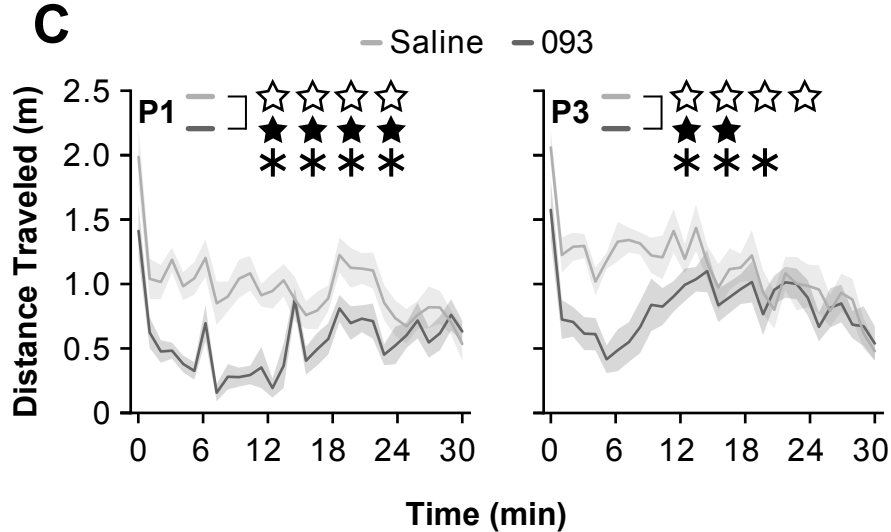

Supplement: Supplementary file 3 — Supplementary Figure 3. 093 causes an acute locomotor deficit that does not produce a conditioned preference or aversion. A) Conditioned place preference for 093 in C57BL/6J mice showed no inherent preference or aversion for the 093‐paired side. B) Animals moved less on days they received 093 (dark grey) compared to saline (light grey). C) Within session locomotion revealed an acute motor deficit reflected by a reduction in distance traveled that recovered within 15‐minutes on 093 days (dark grey) compared to saline days (light grey). Line graphs depict averages ± SEM. Bar graphs show averages ± SEM with points showing individual mice (n = 12, 6 males and 6 females). Empty stars indicate significant main effect of time, filled stars a significant main effect of group, and asterisks a significant main effect of the interaction (n.s. = no significance; * p<0.05; ** p< 0.01; *** p<0.001; **** p<0.0001). [file BRB3-15-e70616-s001.pdf]
